# Insufficient Fruit and Vegetable Intake in a Low- and Middle-Income Setting: A Population-Based Survey in Semi-Urban Tanzania

**DOI:** 10.3390/nu10020222

**Published:** 2018-02-16

**Authors:** Beverly Msambichaka, Ikenna C. Eze, Ramadhani Abdul, Salim Abdulla, Paul Klatser, Marcel Tanner, Ramaiya Kaushik, Eveline Geubbels, Nicole Probst-Hensch

**Affiliations:** 1 Ifakara Health Institute Dar es Salaam, Kiko Avenue, P.O. Box 78373, Dar es Salaam, Tanzania; msambichakabeverly@gmail.com (B.M.); rabdul@ihi.or.tz (R.A.); sabdulla@ihi.or.tz (S.A.); egeubbels@ihi.or.tz (E.G.); 2 Swiss Tropical and Public Health Institute, Socinstrasse 57, 4051 Basel, Switzerland; ikenna.eze@swisstph.ch (I.C.E.); marcel.tanner@swisstph.ch (M.T.); 3 University of Basel, Petersplatz 1, 4001 Basel, Switzerland; 4 Athena Institute, Vrije Universiteit/Free University, 1081 HV Amsterdam, The Netherlands; paulklatser@gmail.com; 5 Shree Hindu Mandal Hospital, Chusi St, P.O. Box 581, Dar es Salaam, Tanzania; ceo@hindumandal.org

**Keywords:** fruit and vegetables, education, occupation, healthcare use, Ifakara, Tanzania

## Abstract

A daily intake of 5 portions of fruit and vegetables (FV) is recommended for protection against non-communicable diseases (NCDs). Inadequate FV intake is a global problem but resource-poor countries like Tanzania are most deprived and constitute settings where little is known for informing public health interventions. This study aimed to describe the prevalence of inadequate FV intake, frequency of FV intake, portions of FV intake and their associations with socio-demographic/lifestyle factors in South-Eastern Tanzania. Data on FV dietary indicators, socio-demographic factors, smoking, alcohol and healthcare use were collected from 7953 participants (≥15 years) of the population-based MZIMA open community cohort (2012–2013). Multivariable logistic regression was used to examine associations between FV intake outcomes and their socio-demographic/lifestyle determinants. Most (82%) of the participants did not meet the recommended daily FV intake While only a fraction consumed fruits daily (15.5%), almost half consumed vegetables daily (44.2%). However, the median (IQR) number of vegetable portions consumed was lower (2(1)/person/day) than that for fruits (2(2)/person/day) People with higher education were more likely to consume fruits daily. Independent correlates of inadequate FV intake included young age, being male, low education, low-income occupations, low alcohol, high tobacco and low healthcare use. Public health interventions should target the socio-economically deprived and culturally-rooted preferences while prioritizing promotion of vegetable for most immediate gain in overall FV intake.

## 1. Introduction

A daily intake of fruits and vegetables (FV) is recommended for protection against almost all major non-communicable diseases (NCDs) [1,2,3,4]. FV have vitamins, minerals and fiber which either singly or synergistically protects against NCDs as well as communicable diseases [4,5]. Antioxidants found in FV can prevent the action of carcinogens by inhibiting oxidative DNA damage [6]. Furthermore, vitamins B12, B6 as well as B9 help reduce levels of homocysteine and risk of cardiovascular diseases (CVDs) [7,8]. Many FV are also rich in potassium which helps modulate blood pressure [9,10]. Maximum benefits from FV could be attained by daily consumption of five portions (400 g) [11,12]. 

Despite promising benefits from FV consumption, more than 75% of the global population do not consume sufficient FV [13] and according to the most recent estimate, this accounted for 2.9% of all lives lost in 2009 [14]. A STEPwise approach to surveillance (STEPS) survey conducted in 2012 showed that almost all Tanzanians (˃95%) consumed insufficient FV [15]. Similar findings were found in neighboring Malawi [16], Zambia [17], Mozambique [18] and Botswana [19]. The STEPS survey findings provide useful information on the scale of the problem but do not allow better characterization of FV intake and susceptibility factors to inadequate FV consumption. Depending on the context, inadequate FV intake may be attributed more to lower fruit intake [20,21], or lower vegetable intake [22]. Hence, it was recently suggested that studies on FV consumption should consider fruits separately from vegetables given that separate interventions may be needed [18,23,24]. According to the literature, most African countries do not have food-based dietary guidelines and the few countries with food based dietary guidelines are not explicit on how much FV should be consumed [25,26,27,28,29]. This is noteworthy because people can only act in favor of good health if they are aware, are convinced and know how to act [30,31]. In Tanzania, the Ministry of Health and Social Welfare promotes two important elements of FV intake, first is daily consumption and second is consumption in high quantities. Shortage of detailed evidence on FV consumption practices hampers targeted responses at policy and health system levels to promote FV consumption [32].

Socio-demographic determinants are important in shaping FV intake patterns [33,34]. Education and wealth, as indicators of socio-economic status (SES) are related to FV intake [13,35]. Education exposes people to necessary health information [36] and builds capacity for comprehension of existing recommendations [31,37,38,39], both of which could be limiting factors when absent [23]. People with more education can secure higher paying jobs which may help address issues of “affordability,” a critical barrier in most developing countries [18,21,40]. Variations in the social, cultural and structural environment of different occupations may also pose different constraints to FV consumption. Lifestyle factors like alcohol consumption and smoking may also in principle affect FV intake where an increased intake may be a compensatory behavior to heavy drinking or smoking [41]. Although this has been explored by Western studies with other lifestyle factors [42,43], evidence from an African population is limited. Although the use of health services may also influence lifestyle choices and vice versa partly through preventive counseling [44], there is limited evidence on how healthcare use influences FV intake. 

Evidence on the importance of socio-demographic determinants in FV consumption may help guide public health responses that are population-specific. In this study, we aimed to describe the patterns of FV consumption and explored how these patterns associate with socio-demographic and lifestyle factors in southeastern Tanzania, using data from the MZIMA open community cohort.

## 2. Materials and Methods

### 2.1. Study Design and Participants

The MZIMA open community cohort is lodged within the Ifakara Urban Health and Demographic Surveillance System (IU-HDSS) [45]. The IU-HDSS is a longitudinal database that collects information on demographic and vital events including births, deaths and migration. The MZIMA cohort was created in 2012 to study among others, changes in NCD burden and their determinants over time [46]. Information collected in the cohort includes socio-demographic characteristics, NCD risk factors such as FV consumption, smoking, alcohol habits as well as health care use. 

Community sensitization activities were conducted and these included meetings with community leaders, pamphlets, radio spots, sensitization at community events. Door-to-door visits, following prior notification by the ten-cell leader, the lowest level of local administration, were conducted. Eligible participants were ≥15 years, willing and able to give informed consent and resident in the study areas during the latest IU-HDSS round. In the first study round, a total of 8734 participants were recruited from June 2012 to April 2013 from two areas of the IU-HDSS (Mlabani and Viwanja Sitini). Community members in the study area are a mix of indigenous inhabitants and migrants. Both Mlabani and Viwanja Sitini are parts of Ifakara ward, situated in Morogoro Region, southeast Tanzania. 

### 2.2. Data Collection

Interviews were conducted at participants’ homes between June 2012 and August 2013 by trained interviewees using a structured questionnaire. The interview tool was translated from English into Swahili and back translated to English and was piloted. Interviewers used tablet personal computers programmed with the open-source Open Data Kit [47]. Automated validation and skip patterns were programmed to minimize faulty data entries. Interviewers also kept field diaries for problems that occurred during data collection. These sheets were reviewed by the supervisor, who made suggestions for improvement, at the end of each day. 

Classification of FV consumption. 

Participants were asked questions on their intake of fruits and intake of vegetables using questions from the WHO STEPS survey tool for NCD risk factors [48]. Questions covered frequency of consumption in a typical week and number of standard portions on days of consumption. A standard portion equals 80 g. Medium size fruits like an orange, an apple, a banana, a pear counted for one portion. Other fruits like half an avocado, half a large mango also formed one standard portion. A typically very large watermelon accounted for 16 portions. One small glass (150 mls) of 100% fruit juice was equal to one portion of fruit. Three heaped tablespoons (~30 g per heaped spoon) of cooked vegetables were equal to one portion. Sometimes people used small bowls for relish, which was equivalent to two standard portions if it were cooked vegetables, or one standard portion if it were fresh salad. Interviewers used picture cards with common fruits and vegetables found in the study setting. The picture cards were used to help participants recall on FV intake in the past week but also to help them minimize errors in estimation of standard portions consumed. In order to get the average daily portions of fruits and portions of vegetables, we multiplied the number of days of consumption and the number of portions consumed in a typical day and divided by seven. Participants who reportedly consumed fruits or vegetables every day in a typical week were classified as having “daily fruit intake” or “daily vegetables intake,” respectively. People who consumed fruits or vegetables on a less than daily basis were categorized as “no daily fruit intake” or “no daily vegetable intake,” respectively. Those without fruit or vegetable intake in a usual week were classified as “no fruit intake” or “no vegetable intake,” respectively. Participants who ate less than 5 portions of fruits and/or vegetables per day were categorized as having inadequate FV intake. 

### 2.3. Covariate Information

Participants were interviewed for their age (years), sex (male/female), marital status (single/monogamous/polygamous/widowed/separated), migration status (migrant/non-migrant), regions of ethnic affiliation (Morogoro/Iringa/Shinyanga/Kilimanjaro/Ruvuma/Coast/Mbeya/Others) and religion (Muslim/Catholic/Lutheran/Others) as previously described [46]. Participants were also asked about their educational attainment and occupation. Educational level was categorized as: no formal education; primary education (up to 7 years of formal education); secondary education (7–13 years of formal education); and tertiary education (>13 years of formal education) [46]. Phrasing for social determinants listed above was adapted from standard questions used in Analyzing Longitudinal Population-based HIV/AIDS data for Africa (ALPHA) network [49]. 

Occupational status was categorized as follows: “unemployed“ being those who have no income generating activity; farming, fishing and livestock keeping; owning a small business (employing < 5 persons); owning a large business (employing ≥ 5 persons); professionals (white collar jobs); skilled manual labors (including drivers, carpenters, etc.); and unskilled manual labors (including menial jobs). Participants were also asked if they smoked or consumed alcohol in the past 12 months [48]. Smoking status was categorized into never, former and current smokers while alcohol use was categorized into daily and not daily, for exploratory purposes, based on the available data. Information on frequency of healthcare visits (hospital, dispensary or home-based care worker) in the past 12 months was also collected and categorized into none, one, two, three, four, five and six or more visits, which allowed for investigating dose-response relationship with FV intake. Participants were also asked if they have been diagnosed with diabetes or hypertension, or any cardiovascular disease in order to derive NCD variable, assigning yes to the presence of any of the three diseases and no to the absence of all three. 

### 2.4. Statistical Analyses

**Figure 1:**
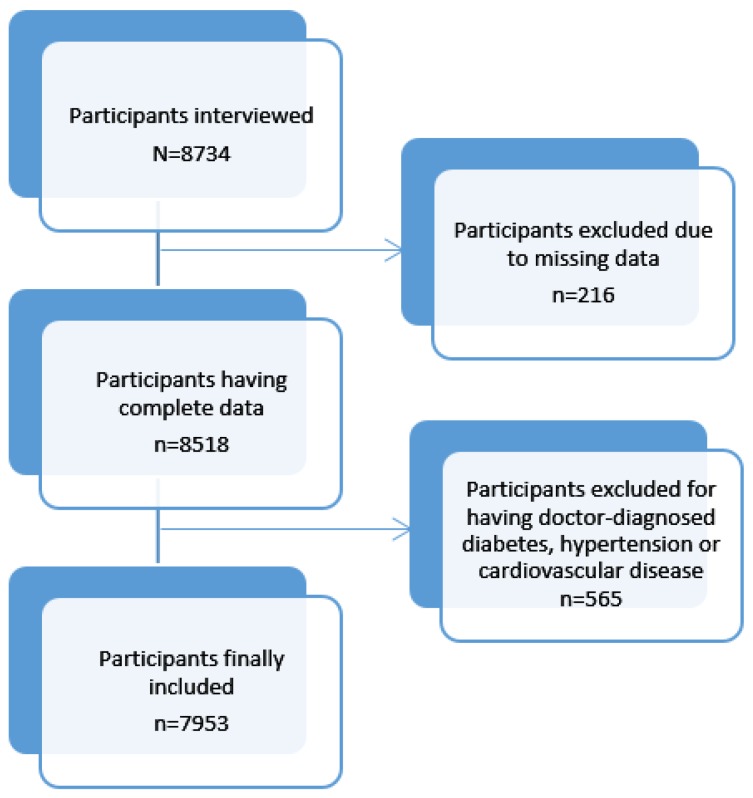
Participant selection flow chart.

We described the study population stratified by sex. We tabulated the FV intake according to socio demographic characteristics and healthcare use. Using three outcome variables- “less than daily fruit intake versus daily fruit intake,” “less than daily vegetable intake versus daily vegetable intake” and “inadequate FV intake versus adequate FV intake,” we applied logistic regression to explore the independent association of these outcome variables with sex, age group, marital status, educational level, occupation, ethnicity, religion and migration status, using mutually-adjusted models. In a further step using the adjusted socio-demographic model, we explored associations of FV intake with lifestyle characteristics and healthcare use habits. Data analyses excluded participants with missing data as well as those with doctor-diagnosed hypertension, diabetes or cardiovascular diseases (Figure 1). The intention for NCD exclusion was to capture trends of FV intake in apparently healthy individuals, towards prevention. All statistical analyses were done using STATA Version 14 (STATA Corporation, Texas). Associations between these outcome variables and socio-demographic and lifestyle determinants are presented as odds ratios (OR) and their 95% confidence intervals. Associations were considered significant at *p* value < 0.05. 

### 2.5. Ethics Consideration

The MZIMA open community cohort was approved by the Ifakara Health Institute Institutional Review Board and the National Institute for Medical Research with reference numbers IHI/IRB/AM/01-2014 and NIMR/HQ/R.8a1Vol. IX/I320 respectively. All participants provided informed written consent to participate in the study. Confidentiality of participants’ identity was ensured by use of encrypted identification codes and proper storage of personal information. 

## 3. Results

### 3.1. Population Description

Out of 8734 (≥15 years) enrolled, 8518 had complete information and 565 participants with confirmed NCDs were excluded bringing the total number of participants for the present analyses to 7953 (Figure 1). Women comprised 64.3% of the study population. Participants below 18 years comprised 27% of those below 25 years and 10% of the entire study population. More than half (55.2%) of the participants were educated at primary level and more women than men had not received any formal education. More than half of all participants were engaged in an income-generating activity (59.8%) and were mainly farmers (25.4%). Major ethnic groups included Morogoro, Iringa and Ruvuma. Alcohol consumption and smoking rates were generally low. (Table 1).

### 3.2. Patterns of FV Consumption

Inadequate FV consumption was observed in 82% of the study population (Table 2), i.e., the prevalence of not meeting the recommendation for daily eating of fruits or vegetables. Fruits tended to be consumed less frequently than vegetables. However, median vegetable portions were smaller than median portions of fruits (Table 2). Inadequate FV consumption appeared less prevalent as people became more educated, as well as in people who have higher-earning occupations. Inadequate FV consumption also tended to decrease with increasing use of healthcare services (Table 2). 

Almost the entire study population consumed at least some vegetables (98.5%) in the seven days preceding the interview. Vegetable consumption revealed two main patterns; “Daily vegetable intake” (44.2%) and “No daily vegetable intake” (54.3%). Median (IQR) vegetable portions consumed per day per person was 2 (1) portions. Older participants tended to have higher number of standard vegetable portions than younger people. The frequency of daily vegetable intake appeared to be lowest among least educated, unskilled and skilled laborers. Participants with more frequent use of healthcare also tended to have higher proportion of daily vegetable consumption when compared to those with less frequent health care use (Table 2). 

**Figure 2:**
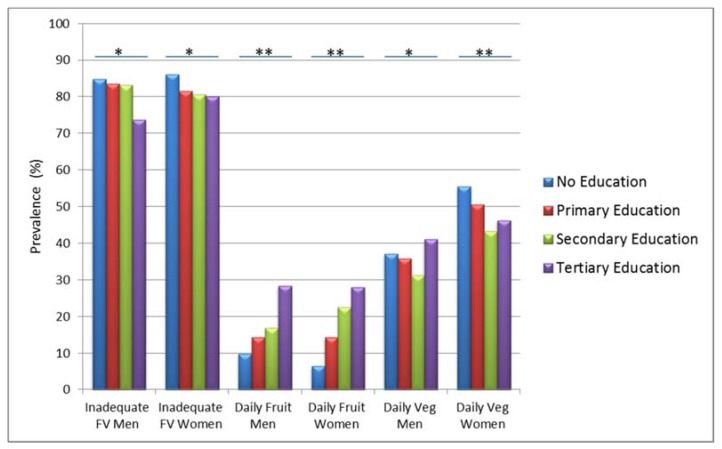
Prevalence of daily fruit, daily vegetable and inadequate fruits and vegetables (FV) intake among men and women in different education categories (*N* = 7953). * Significant differences in fruit or vegetable intake across different educational levels (*p* < 0.05); ** Significant differences in fruit or vegetable intake across different educational levels (*p* < 0.0001).

The median (IQR) fruit portions consumed per day per person was 2 (2) portions. For fruits, three patterns emerged “Daily fruit intake” (15.5%), “No daily fruit intake” (71.7%) and “No fruit intake” (12.7%). Younger participants appeared to consume more fruits daily (16.1%) compared to older participants (8.1%). The oldest age group had the lowest median (IQR) fruit portions (1 (1) portions). Figure 2 shows that participants tended to consume more FV with higher educational level, irrespective of gender.

### 3.3. Independent Association of FV Intake with Socio-demographic Characteristics

Women were at a lesser risk for less than daily fruit (OR = 0.84, 95% CI: 0.73, 0.96) and vegetable intake (OR = 0.51, 95% CI: 0.46, 0.56) as well as inadequate FV (OR = 0.82, 95% CI: 0.72, 0.93) compared to men (Table 3). There was a strong association between the risk of less than daily vegetable intake and age. Older participants were less likely to have less than daily vegetable intake with OR of 0.54 (95% CI: 0.43, 0.67) for the oldest age group compared to the youngest age group. Lower education was significantly associated with less than daily fruit intake but not vegetable intake. Odds of less than daily fruit intake decreased in those with primary education (OR = 0.55, 95% CI: 0.43, 0.70) and those with secondary education (OR = 0.27, 95% CI: 0.18, 0.40) compared to those without any formal education. Employment and occupation were important in the overall risk of inadequate FV intake. Those with higher earning occupations like small (OR = 0.83, 95% CI: 0.69, 1.00), large business owners (OR = 0.50, 95% CI: 0.32, 0.83) and professionals (OR = 0.66, 95% CI: 0.50, 0.86) were less likely to have inadequate FV intake compared to farmers. Migrants were at higher risk of less than daily fruit consumption as well as overall inadequate FV intake compared to non-migrants. Also, ethnic groups from Iringa (OR = 1.31, 95% CI: 1.12, 1.52), Shinyanga/Mwanza/Tabora (OR = 1.78, 95% CI: 1.45, 2.21), Kilimanjaro (OR = 1.39, 95% CI: 1.06, 1.82) and Mbeya (OR = 1.79, 95% CI: 1.20, 2.64) were more likely not to consume vegetables daily compared to those from Morogoro but there were no significant differences in their overall FV intake (Table 3). 

Participants who reported drinking alcohol on a daily basis were less at risk for less than daily fruit (OR = 0.68, CI: 0.47, 0.98), vegetable (OR = 0.68, CI: 0.50, 0.92) and inadequate FV consumption (OR = 0.62, CI: 0.44, 0.86) even after adjustment for all socio-demographic indicators and this was similar for men and women (Table 4). In contrast, no statistically-significant associations were observed between FV consumption and smoking. 

**Figure 3:**
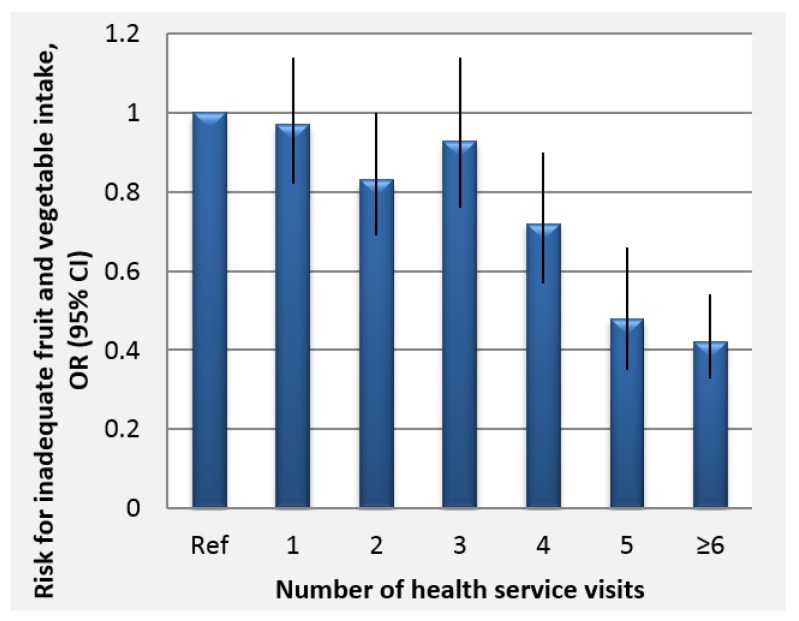
Odds ratios and confidence intervals for the association of inadequate fruit and vegetable intake with frequency of healthcare use in the previous 12 months (cumulate number of outpatient, health dispensary and home visits) *N* = 7953.

We observed a strong association between inadequate FV intake and healthcare use in this cross-sectional study even after adjusting for various socio-demographic factors as well as smoking and alcohol consumption. . Higher healthcare service use seemed to be protective for inadequate FV intake. The results showed maximum protection for participants who reported ≥ 6 visits where the odds of inadequate FV intake reduced by 59% compared to those without any healthcare visits (Figure 3). We did not observe any sex differences in the association between inadequate FV intake and healthcare use (result not shown). 

## 4. Discussion

In line with findings from other STEPs surveys done in the sub-Saharan African region [15,16,17,18,19], most of these rural and semi-urban Tanzanian study participants did not meet the five portions daily FV consumption recommendation. More effort is needed to increase the frequency of fruit intake than is needed to increase the frequency of vegetable intake. This is because almost half of the participants consumed vegetables daily while a lesser fraction consumed fruits daily. An important target group for nutritional recommendation is persons of lower SES. 

We generally observed that vegetables are widely consumed but in very small portions while fruits are mainly consumed in larger portions, especially among those with higher SES. Daily vegetable consumption was three-fold more prevalent when compared to daily fruit intake. Furthermore, those who had not eaten a fruit at all in the past 7 days of the interview were almost ten times more (12.7%) than those who had not eaten vegetables (1.5%) at all. Our finding of a higher consumption of vegetables than fruits is supported by evidence from studies in South Africa and Mozambique [18,20]. Vegetables are a cheap relish that accompanies daily staples like stiff polenta (“Ugali”), rice, flat bread and others [21,50] and this may explain to a large extent, the high daily vegetable consumption. 

Although inadequate FV intake was not different between men and women, it was evident in our findings that men were 49% less likely to consume vegetables daily. This could be due to gender differences in health consciousness or gender roles which may give women more access to food supplies [51,52,53] as well as the fact that men tend to consume more out of home prepared meals especially for lunch while at work [54], most of which is low on vegetables. 

Epidemiologic transition in the semi-urban settings where particularly younger people are exposed to more western-style diets may explain the observed decline of daily vegetable intake as well as portions sizes with decreasing age. Although participants under 18 years are more likely to be financially dependent, thus depending on parental food choices, the observed protective age effect was also made among subjects older than 25 years. This age group (<18 years) comprised only 10% of our study sample, thus, we expect minimal bias of our results due to their inclusion. In rural settings, older people have shown more attachment to vegetables as their source of nutrition, being that they are mostly farmers with a wealth of knowledge on vegetable varieties and use [50,55]. Interestingly, in this study we did not observe farmers to have high vegetable intake. 

For fruit intake, cost is a clear barrier in many poor settings similar to Tanzania [56,57]. Our findings demonstrate the importance of SES to fruit intake. Higher level of education and high-income occupations were facilitators of more frequent and larger portions of fruit consumption. It has been previously reported that affordability of fruits is an important bottleneck for their consumption [18,20,54]. Unlike vegetables, the relationship between income and affordability of fruits has received global attention [13,56]. The relative cost of fruits in low income countries has been reported to be 50 times higher than the relative costs in high income countries [58]. Fruits are easy-to-perish commodities and often lack controlled environments for quality storage, transportation as well as packing. This leads to large post-harvest losses and results in high market prices of fruits [59]. 

Cultural perceptions on FV intake differ. Contrary to our findings on vegetable intake, the oldest participants consumed least fruits. This may be explained by attitudes towards fruits as demonstrated by another study in neighboring Zanzibar where fruits are regarded as snacks for children [22,60]. Another study conducted in rural Tanzania, also reported that children consumed fruits the most [61]. The difference in association between fruit intake and age groups across countries in different continents [62,63,64] suggests culture to be an important context that needs to be considered in developing prevention programs.

This study also found that frequent health care use was associated with more FV consumption. Even though we do not have information on reasons for health care use, it is more likely that those with more health care use are more health conscious and may therefore benefit from preventive health advice including FV consumption. Exploratory analyses suggested that participants with higher SES were more likely to have higher healthcare use thus, health education or promotions aimed at people most vulnerable to inadequate FV intake, should rely more on community-based approaches rather than health facilities as the delivery channel. This, in addition, provides an opportunity for public health interventions to address both inadequate FV intake and poor health service use through integrated approaches. Our observation of higher consumption of FV among those who drank more alcohol is supported by previous studies which showed that individuals tend to compensate an unhealthy lifestyle with another healthy one [41]. Interestingly, this was not true for smoking in our study. 

### Strengths and Limitations

The strengths of this study derive from being the first study to the best of our knowledge from Tanzania providing detailed description about patterns of FV intake and socio-demographic and lifestyle determinants. This study also contributes in detail, to the growing literature on the burden of NCD risk factors in Africa. Our sample size is large and representative of the IU-HDSS area. The MZIMA cohort includes 70% of eligible adults from the HDSS area [46] whereas our sample includes 92% of the MZIMA cohort. All ethnic groups, religious affiliations and other socio-demographic attributes of the area were well-captured in our sample. We explored FV intake both separately and in combination according to WHO recommendations. The diverse cultural make-up makes these findings generalizable to other small towns with similar settings. Being nested within the IU-HDSS, a part of the INDEPTH network and as the NCD research agenda is building up in the African continent, our findings will make a useful source of comparison to similar studies in the future in Tanzania and beyond. Our study also has limitations. First, it was a cross-sectional study and precludes drawing conclusions about causal associations. Second, there may have been recall bias in the responses for FV intake. However, the recall period was short (seven days) hence, we expect minimal recall bias. The short recall period in this setting is also likely representative of longer-term dietary habits. Second, there was lack of information on some of the possible confounders or modifiers, including awareness about importance of FV intake, actual purchasing power and availability. These findings represent data collected over one year and no specific seasonal analysis was done. 

## 5. Conclusions

FV consumption in the study setting was associated with SES and cultural patterns. Most immediate gain in improving overall FV consumption in resource-poor settings may be attained by promoting daily consumption of vegetables and increase in number of standard portions of vegetables consumed. Improving access to fruits by making them more affordable may contribute to improving intake rates. More qualitative and quantitative research are needed to better understand the prevalent knowledge, attitude and perception of fruits and vegetable consumption in local cultural contexts in order to improve their intake rates in these settings. Given the importance of awareness in health behavior change, more effort is also needed in the dissemination of the message regarding the number of FV portions necessary to maintain good health.

## Figures and Tables

**Table 1:** Description of the study population.

Variables	Groups	All % (*N*)	Males % (*N*)	Females % (*N*)	Chi- Squared Test
All participants		100 (7953)	35.7(2839)	64.3 (5114)	N/A
Age	Below 25 years	39.1 (3111)	36.2 (1027)	40.8 (2084)	<0.001
	25–50 years	45.1 (3588)	45.1 (1292)	44.9 (2296)	
	50–60 years	6.7 (535)	7.3 (206)	6.4 (329)	
	60 and above	9.0 (719)	11.1 (314)	7.9 (405)	
Education	No Formal Education	14.1 (1118)	9.1 (258)	16.8 (860)	<0.001
	Primary Education	55.2 (4387)	53.0 (1505)	56.4 (2882)	
	Secondary Education	27.5 (2186)	32.9 (935)	24.5 (1251)	
	Tertiary Education	3.3 (262)	5.0 (141)	2.4 (121)	
Marriage	Never married	38.8 (3088)	46.6 (1324)	34.5 (1764)	<0.001
	Monogamous	47.4 (3770)	44.7 (1268)	48.9 (2502)	
	Polygamous	1.2 (94)	1.1 (30)	1.3 (64)	
	Widowed	5.8 (461)	2.4 (68)	7.7 (393)	
	Separated	6.8 (540)	5.3 (149)	7.7 (391)	
Work status	Working	59.8 (4754)	72.1 (2047)	52.9 (2707)	<0.001
	Not working	40.2 (3199)	27.9 (792)	47.1 (2407)	
Occupation	Farming, Fishing, Livestock keeping	25.4 (2017)	26.9 (763)	24.5 (1254)	<0.001
	Small business	15.4 (1221)	14.8 (421)	15.6 (800)	
	Large business	1.2 (95)	2.3 (66)	0.6 (29)	
	Professionals	4.7 (367)	6.5 (183)	3.6 (184)	
	Skilled manual labor	7.9 (625)	12.4 (352)	5.3 (273)	
	Unskilled manual labor	5.4 (429)	9.2 (262)	3.3 (167)	
	Not working	40.2 (3199)	27.9 (792)	47.1 (2407)	
Religion	Muslim	37.1 (2952)	38.2 (1085)	36.5 (1867)	0.258
	Catholic	54.3 (4317)	53.6 (1521)	54.7 (2796)	
	Lutheran	1.9 (147)	1.6 (44)	2.0 (103)	
	Other beliefs	6.8 (537)	6.7 (189)	6.8 (348)	
Migration	Non-migrant	41.5 (3301)	40.4 (1146)	42.1 (2155)	0.124
	Migrant	58.5 (4652)	59.6 (1693)	57.9 (2959)	
Ethnicity	Mbeya region	1.6 (127)	1.4 (41)	1.7 (86)	<0.001
	Kilimanjaro and Arusha region	3.3 (265)	4.1 (115)	2.9 (150)	
	Coast region	6.2 (491)	6.2 (177)	6.1 (314)	
	Shinyanga/Mwanza/Tabora regions	6.9 (545)	8.2 (234)	6.1 (311)	
	Iringa region	11.4 (904)	10.6 (302)	11.8 (602)	
	Ruvuma region	14.8 (1179)	14.2 (403)	15.2 (776)	
	Other regions	12.8 (1020)	14.7 (418)	11.8 (602)	
	Morogoro region	43.0 (3422)	40.5 (1147)	44.5 (2273)	
Alcohol	Not daily	97.6 (7764)	96.7 (2746)	98.1 (5018)	<0.001
	Daily	2.4 (189)	3.3 (93)	1.9 (96)	
Smoking	Never smoker	89.7 (7131)	76.4 (2169)	97.0 (4962)	<0.001
	Former smoker	3.1 (245)	6.5 (183)	1.2 (62)	
	Current smoker	7.3 (577)	17.2 (487)	1.8 (90)	
Healthcare use	No visit	40.0 (3180)	45.5 (1291)	36.9 (1889)	<0.001
	One visit	20.7 (1643)	21.3 (604)	20.3 (1039)	
	Two visits	12.6 (1004)	11.6 (328)	13.2 (676)	
	Three visits	11.8 (935)	10.7 (305)	12.3 (630)	
	Four visits	7.4 (590)	5.5 (157)	8.5 (433)	
	Five visits	2.7 (214)	2.3 (64)	2.9 (150)	
	Six visits and more	4.9 (387)	3.2 (90)	5.8 (297)	

N/A: not applicable. The chi-squared test compares proportions between males and females.

**Table 2:** Frequency and Patterns of Fruit and Vegetable Intake in the MZIMA Cohort, *N* = 7953.

Variable	Groups	Daily Fruit Intake ^a^ % (*N*)	No Daily Fruit Intake ^b^ % (*N*)	No Fruit Intake ^c^ % (*N*)	Daily Fruit Portions (Median (IQR))	Daily Vegetable Intake ^a^ % (*N*)	No Daily Vegetable Intake ^b^ % (*N*)	No Vegetable Intake ^c^ % (*N*)	Daily Vegetable Portions (Median (IQR))	Inadequate FV Intake ^d^ (%)	Chi Squared Test
All	N/A	15.4 (1227)	72.1 (5734)	12.5 (992)	2 (2)	44.2 (3516)	54.3 (4318)	1.5 (119)	2 (1)	82	N/A
Sex	Male	15.6 (440)	72.7 (2064)	11.8 (335)	2 (2)	34.7 (985)	62.8 (1782)	2.5 (72)	1 (1)	83	0.269
	Female	15.4 (787)	71.8 (3670)	12.9 (657)	2 (2)	49.5 (2531)	49.6 (2536)	0.9 (47)	2 (1)	82	
Age	Below 25	16.1 (502)	72.5 (2254)	11.4 (355)	2 (2)	36.1 (1124)	62.1 (1932)	1.8 (55)	1 (1)	84	<0.001
	25–50	15.9 (572)	72.6 (2603)	11.5 (413)	2 (2)	46.4 (1665)	52.1 (1872)	1.4 (51)	2 (1)	82	
	50–60	17.8 (95)	65.6 (351)	16.6 (89)	2 (2)	55.2 (397)	43.7 (314)	0.9 (5)	2 (1)	76	
	Above 60	8.1 (58)	73.2 (526)	18.8 (135)	1 (1)	55.2 (397)	43.7 (314)	1.1 (8)	2 (1)	84	
Education	No Education	7.3 (81)	71.1 (795)	21.7 (242)	1 (1)	51.3 (573)	47.1 (527)	1.6 (18)	2 (1)	86	0.002
	Primary	14.4 (631)	73.7 (3231)	12.0 (525)	2 (2)	45.5 (1996)	53.3 (2336)	1.3 (55)	2 (1)	82	
	Secondary	20.2 (441)	70.3 (1537)	9.5 (208)	2 (2)	38.1 (883)	60.0 (1312)	1.9 (41)	1 (1)	82	
	Tertiary	28.2 (74)	65.3 (171)	6.5 (17)	2 (2)	43.5 (114)	54.6 (143)	1.9 (5)	1 (1)	77	
Marital status	Never married	15.7 (485)	72.6 (2243)	11.7 (360)	2 (2)	36.2 (1117)	61.7 (1904)	2.2 (67)	1 (1)	84	0.001
	Monogamous	16.3 (627)	72.1 (2718)	11.3 (425)	2 (2)	47.6 (1796)	51.4 (1937)	1.0 (37)	2 (1)	81	
	Polygamous	14.9 (14)	69.2 (65)	16.0 (15)	2 (2)	55.3 (52)	43.6 (41)	1.1 (1)	2 (1)	79	
	Widowed	8.0 (37)	69.2 (319)	22.8 (105)	1 (1)	59.4 (274)	39.3 (181)	1.3 (6)	2 (1)	84	
	Divorced	11.9 (64)	72.0 (389)	16.1 (87)	1 (2)	51.3 (277)	47.2 (255)	1.5 (8)	2 (1)	85	
Work status	Working	16.4 (780)	71.8 (3415)	11.8 (559)	2 (2)	47.4 (2252)	51.3 (2437)	1.4 (65)	2 (1)	80	<0.001
	Not working	14.0 (447)	72.5 (2319)	13.5 (433)	2 (2)	39.5 (1264)	58.8 (1881)	1.7 (54)	2 (1)	85	
Occupation	Farming, Fishing, Livestock	12.0 (241)	75.8 (1528)	12.3 (248)	2 (2)	53.1 (1070)	46.0 (928)	0.9 (19)	2 (1)	82	<0.001
	Small business	18.0 (221)	71.7 (876)	10.2 (124)	2 (2)	45.5 (556)	53.2 (649)	1.3 (16)	2 (1)	79	
	Large business	30.5 (29)	62.1 (59)	7.4 (7)	2 (3)	47.4 (45)	49.5 (47)	3.2 (3)	1 (1)	71	
	Professionals	28.6 (105)	64.0 (235)	7.4 (27)	2 (3)	47.7 (175)	52.0 (191)	0.3 (1)	1 (1)	73	
	Skilled manual labor	20.4 (129)	66.9 (418)	12.5 (78)	2 (2)	40.0 (250)	58.7 (367)	1.3 (8)	2 (1)	80	
	Unskilled manual labor	12.8 (55)	66.7 (299)	17.5 (7)	2 (2)	36.4 (156)	59.4 (255)	4.2 (18)	2 (1)	87	
	Not working	14.0 (447)	72.5 (2319)	13.5 (433)	2 (2)	39.5 (1264)	58.8 (1881)	1.7 (54)	2 (1)	85	
Religion	Muslim	15.6 (459)	71.8 (2119)	12.7 (374)	2 (2)	44.7 (1319)	53.6 (1582)	1.7 (51)	2 (1)	82	0.934
	Catholic	14.9 (641)	72.8 (3141)	12.4 (535)	2 (2)	44.5 (1922)	54.3 (2345)	1.2 (50)	1 (1)	83	
	Lutheran	24.5 (36)	60.5 (89)	15.0 (22)	2 (2)	49.0 (72)	50.3 (74)	0.7 (1)	1 (1)	84	
	Others	17.0 (91)	71.7 (385)	11.4 (61)	2 (2)	37.8 (203)	59.0 (317)	3.2 (17)	1 (1)	83	
Migration	Non-migrants	16.0 (527)	71.4 (2357)	12.6 (417)	2 (2)	44.8 (1478)	54.2 (1788)	1.1 (35)	2 (1)	82	0.151
	Migrants	15.0 (700)	72.6 (3377)	12.4 (575)	2 (2)	43.8 (2038)	54.4 (2530)	1.8 (84)	2 (1)	83	
Ethnicity	Morogoro region	14.2 (485)	72.2 (2471)	13.6 (466)	2 (2)	48.6 (1664)	50.4 (1723)	1 (35)	2 (1)	82	0.140
	Iringa region	14.6 (132)	73.8 (667)	11.6 (105)	2 (2)	41.0 (371)	58.2 (526)	0.7 (7)	1 (1)	83	
	Shinyanga/Mwanza/Tabora	13.9 (76)	76.2 (415)	9.9 (54)	2 (2)	30.8 (168)	65.5 (357)	3.7 (20)	2 (1)	85	
	Kilimanjaro/Arusha region	24.2 (64)	67.6 (179)	8.3 (22)	2 (3)	37.4 (99)	60.4 (160)	2.3 (6)	2 (1)	78	
	Ruvuma region	15.4 (181)	72.2 (851)	12.5 (147)	2 (2)	45.3 (534)	53.7 (633)	1 (12)	1 (1)	81	
	Coast region	14.0 (71)	70.9 (348)	14.7 (72)	2 (2)	45.4 (223)	52.6 (258)	2.0 (10)	2 (1)	84	
	Mbeya region	17.3 (22)	74.8 (95)	7.9 (10)	2 (2)	32.3 (41)	66.1 (84)	1.6 (2)	2 (1)	87	
	Other regions	19.2 (196)	69.4 (708)	11.4 (116)	2 (2)	40.8 (416)	56.8 (577)	2.7 (27)	1 (1)	83	
Alcohol use	Not Daily	15.3 (1187)	72.3 (5615)	12.4 (962)	2 (2)	43.9 (3409)	54.6 (4239)	1.5 (116)	2 (1)	83	<0.001
	Daily	21.2 (40)	63.0 (119)	15.9 (30)	2 (3)	56.6 (107)	41.8 (79)	1.6 (3)	2 (1)	72	
Smoking	Never	15.7 (1119)	72.5 (5167)	11.9 (845)	2 (2)	44.4 (3164)	54.3 (3870)	1.4 (97)	1 (1)	82	0.630
	Former	12.2 (30)	71.8 (176)	15.9 (39)	2 (2)	46.9 (115)	49.0 (120)	4.1 (10)	2 (1)	81	
	Current	13.5 (78)	67.8 (391)	18.7 (108)	2 (2)	41.1 (237)	56.9 (328)	2.1 (12)	2 (1)	84	
Healthcare use	No visits	12.8 (407)	73.0 (2322)	14.2 (451)	2 (2)	38.0 (1208)	60.4 (1919)	1.7 (53)	2 (1)	85	<0.001
	One visit	16 (259)	72.4 (1190)	11.8 (194)	2 (2)	44.2 (726)	54.2 (890)	1.6 (27)	2 (1)	84	
	Two visits	16.4 (165)	71.1 (714)	12.5 (125)	2 (2)	47.7 (479)	51.1 (513)	1.2 (12)	1 (1)	82	
	Three visits	16.7 (156)	73.2 (684)	10.2 (95)	2 (2)	48.3 (452)	50.7 (474)	1.0 (9)	2 (1)	83	
	Four visits	18.0 (106)	73.2 (432)	8.8 (52)	2 (2)	50.7 (299)	48.0 (283)	1.4 (8)	1 (1)	79	
	Five visits	19.2 (41)	65.4 (140)	15.4 (33)	2 (3)	53.7 (115)	44.9 (96)	1.4 (3)	1 (1)	72	
	Six visits and more	24.0 (93)	65.1 (252)	10.6 (42)	2 (3)	61.2 (237)	37.0 (143)	1.8 (7)	1 (1)	69	

IQR: interquartile range. N/A: not applicable. The chi-squared test refers to the comparison of inadequate FV intake across categories of socio-demographic and lifestyle variables; ^a^ Participants who reported daily consumption; ^b^ Participants who reported consumption on a less than daily basis; ^c^ Participants who reported no consumption; ^d^ Participants who reported consumption of less than 5 portions of fruits and/or vegetables per day.

**Table 3:** Association of fruit and vegetable intake with socio-demographic characteristics (*N* = 7953).

		Risk for Less than Daily Fruit Intake		Risk for Less than Daily Vegetable Intake		Risk for Inadequate Fruit and Vegetable Intake	
		OR *	95% CI	OR **	95% CI	OR ***	95% CI
**Sex**	Men	Ref	-	Ref	-	Ref	-
	Women	**0.84**	**0.73–0.96**	**0.51**	**0.46–0.56**	**0.82**	**0.72–0.93**
**Age**	<25 years	Ref	-	Ref	-	Ref	-
	25–50	1.00	0.85–1.20	**0.80**	**0.70–0.91**	**0.94**	**0.79–1.11**
	50–60	**0.70**	**0.51–0.93**	**0.46**	**0.37–0.57**	**0.60**	**0.46–0.79**
	>60	1.28	0.88–1.76	**0.54**	**0.43–0.67**	0.79	0.59–1.05
**Marital Status**	Never married/cohabiting	Ref	-	Ref	-	Ref	-
	Monogamously married/cohabiting	**0.84**	**0.71–0.99**	**0.88**	**0.77–0.99**	0.89	0.76–1.05
	Polygamous married/cohabiting	0.90	0.49–1.65	0.75	0.48–1.15	0.84	0.50–1.43
	Widowed	1.33	0.88–2.01	0.86	0.67–1.11	1.19	0.85–1.66
	Separated/divorced	1.13	0.83–1.54	0.88	0.72–1.09	1.21	0.91–1.59
**Education Level**	No education	Ref	-	Ref	-	Ref	-
	Primary	**0.55**	**0.43–0.70**	1.01	0.87–1.17	**0.78**	**0.64–0.95**
	Secondary	**0.35**	**0.27–0.46**	1.00	0.85–1.19	**0.67**	**0.53–0.84**
	Tertiary	**0.27**	**0.19–0.40**	0.94	0.70–1.27	0.61	0.43–0.88
**Occupation**	Farming/Livestock/Fishing	Ref	-	Ref	-	Ref	-
	Small business	**0.69**	**0.56–0.84**	1.16	1.00–1.34	**0.84**	**0.69–1.00**
	Large business	**0.39**	**0.25–0.63**	0.75	0.49–1.15	**0.50**	**0.32–0.83**
	Professionals	**0.51**	**0.39–0.68**	1.00	0.79–1.28	**0.66**	**0.50–0.86**
	Skilled manual workers & drivers	**0.61**	**0.48–0.78**	**1.25**	**1.03–1.51**	0.87	0.68–1.09
	Unskilled laborers & bar workers	0.90	0.65–1.25	**1.28**	**1.02–1.60**	1.26	0.92–1.72
	Not working	0.99	0.81–1.20	**1.46**	**1.27–1.66**	**1.28**	**1.07–1.52**
**Ethnicity**	Morogoro	Ref	-	Ref	-	Ref	-
	Iringa	1.03	0.83–1.28	**1.31**	**1.12–1.52**	1.08	0.88–1.32
	Shinyanga/Mwanza/Tabora	1.06	0.80–1.41	**1.78**	**1.45–2.21**	1.14	0.87–1.49
	Kilimanjaro	**0.66**	**0.48–0.91**	**1.39**	**1.06–1.82**	0.83	0.60–1.14
	Ruvuma	1.98	0.81–1.18	1.09	0.95–1.25	0.94	0.79–.12
	Coast	0.96	0.72–1.27	1.06	0.87–1.30	1.13	0.86–1.47
	Mbeya	0.91	0.56–1.48	**1.79**	**1.20–2.64**	1.43	0.83–2.46
	Other	**0.79**	**0.65–0.96**	**1.18**	**1.01–1.37**	1.07	0.88–1.31
**Religion**	Muslim	Ref	-	Ref	-	Ref	-
	Catholic	1.12	0.97–1.28	0.97	0.88–1.08	1.06	0.93–1.21
	Lutheran	0.69	0.46–1.03	0.68	0.48–0.97	1.12	0.70–1.77
	Other & No Religion	0.99	0.75–1.29	1.02	0.83–1.26	0.98	0.75–1.27
**Migration Status**	Non-Migrant	Ref	-	Ref	-	Ref	-
	Migrant	**1.15**	**1.00–1.31**	1.08	0.97–1.19	**1.14**	**1.00–1.30**

All estimates were from a multivariable model adjusting for gender, age, marital status, educational level, occupation, ethnicity, religion and migration status. * OR > 1 and OR < 1, describes the increased and decreased likelihood to consume fruits less than daily respectively; ** OR > 1 and OR < 1, describes the increased and decreased likelihood to consume vegetables less than daily respectively; *** OR > 1 and OR < 1, describes the increased and decreased likelihood to consume less than 5 portions fruits and vegetables daily respectively.

**Table 4:** Association of inadequate fruit and vegetable intake with smoking and alcohol consumption (*N* = 7953).

			Risk for Less than Daily Fruit Intake		Risk for Less than Daily Vegetable Intake		Risk for Inadequate Fruit and Vegetable Intake	
			OR *	95% CI	OR **	95% CI	OR ***	95% CI
**All subjects**	Smoking status	Never	Ref	-	Ref	-	Ref	-
		Former	1.1	0.70–1.61	0.98	0.74–1.29	0.91	0.64–1.28
		Current	1.00	0.76–1.31	0.68	0.50–0.92	1.05	0.82–1.35
	Alcohol consumption	Not daily	Ref	-	Ref	-	Ref	-
		Daily	**0.68**	**0.47–0.98**	**0.68**	**0.50–0.92**	**0.62**	**0.44–0.86**
**Men**	Smoking status	Never	Ref	-	Ref	-	Ref	-
		Former	0.96	0.62–1.51	0.85	0.61–1.18	0.84	0.57–1.26
		Current	1.01	0.75–1.36	0.94	0.75–1.17	0.97	0.74–1.20
	Alcohol consumption	Not daily	Ref	-	Ref	-	Ref	-
		Daily	0.85	0.45–1.47	**0.59**	**0.38–0.90**	0.83	0.50–1.39
**Women**	Smoking status	Never	Ref	-	Ref	-	Ref	-
		Former	3.36	0.79–14.32	1.39	0.82–2.35	1.19	0.57–2.49
		Current	1.52	0.64–3.59	1.17	0.75–1.82	1.83	0.90–3.73
	Alcohol consumption	Not daily	Ref	-	Ref	-	Ref	-
		Daily	**0.57**	**0.35–0.95**	0.79	0.52–1.20	**0.48**	**0.31–0.74**

All estimates were from a mutually-adjusted model, additionally adjusted for socio-demographic characteristics (gender, age, marriage, education, occupation, ethnicity, religion and migration). * OR > 1 and OR < 1, describes the increased and decreased likelihood to consume fruits less than daily respectively; ** OR > 1 and OR < 1, describes the increased and decreased likelihood to consume vegetables less than daily respectively; *** OR > 1 and OR < 1, describes the increased and decreased likelihood to consume less than 5 portions fruits and vegetables daily respectively.
